# Pentoxifylline alleviates ischemic white matter injury through up-regulating Mertk-mediated myelin clearance

**DOI:** 10.1186/s12974-022-02480-4

**Published:** 2022-05-31

**Authors:** Lili Zheng, Junqiu Jia, Yan Chen, Renyuan Liu, Runjing Cao, Manlin Duan, Meijuan Zhang, Yun Xu

**Affiliations:** 1 Department of Neurology, Drum Tower Hospital, Medical School and The State Key Laboratory of Pharmaceutical Biotechnology, Institute of Brain Science, Nanjing University, Nanjing University Medical School, 321 ZhongShan Road, Nanjing, 210008 Jiangsu China; 2 Jiangsu Key Laboratory for Molecular Medicine, Medical School of Nanjing University, Nanjing, China; 3 Jiangsu Province Stroke Center for Diagnosis and Therapy, Nanjing, China; 4 Nanjing Neuropsychiatry Clinic Medical Center, Nanjing, China; 5 Department of Anesthesiology, Affiliated Jinling Hospital, Medical School of Nanjing University, Nanjing, China; 6 Department of Radiology, The Affiliated Drum Tower Hospital, Nanjing University Medical School, Nanjing, China

**Keywords:** Vascular dementia, Pentoxifylline, Mertk, PPAR-γ, Phagocytosis, Microglia

## Abstract

**Background:**

Vascular dementia (VAD) is the second most common type of dementia lacking effective treatments. Pentoxifylline (PTX), a nonselective phosphodiesterase inhibitor, displays protective effects in multiple cerebral diseases. In this study, we aimed to investigate the therapeutic effects and potential mechanisms of PTX in VAD.

**Methods:**

Bilateral common carotid artery stenosis (BCAS) mouse model was established to mimic VAD. Mouse behavior was tested by open field test, novel object recognition test, Y-maze and Morris water maze (MWM) tests. Histological staining, magnetic resonance imaging (MRI) and electron microscopy were used to define white matter integrity. The impact of PTX on microglia phagocytosis, peroxisome proliferator-activated receptors-γ (PPAR-γ) activation and Mer receptor tyrosine kinase (Mertk) expression was assessed by immunofluorescence, western blotting and flow cytometry with the application of microglia-specific Mertk knockout mice, Mertk inhibitor and PPAR-γ inhibitor.

**Results:**

Here, we found that PTX treatment alleviated cognitive impairment in novel object recognition test, Y-maze and Morris water maze tests. Furthermore, PTX alleviated white matter injury in corpus callosum (CC) and internal capsule (IC) areas as shown by histological staining and MRI analysis. PTX-treatment group presented thicker myelin sheath than vehicle group by electron microscopy. Mechanistically, PTX facilitated microglial phagocytosis of myelin debris by up-regulating the expression of Mertk in BCAS model and primary cultured microglia. Importantly, microglia-specific Mertk knockout blocked the therapeutic effects of PTX in BCAS model. Moreover, Mertk expression was regulated by the nuclear translocation of PPAR-γ. Through modulating PPAR-γ, PTX enhanced Mertk expression.

**Conclusions:**

Collectively, our results demonstrated that PTX showed therapeutic potentials in VAD and alleviated ischemic white matter injury via modulating Mertk-mediated myelin clearance in microglia.

**Supplementary Information:**

The online version contains supplementary material available at 10.1186/s12974-022-02480-4.

## Introduction

Vascular dementia (VAD) has been the second common type of dementia and lack of effective treatments [1, 2]. Chronic hypoperfusion leads to white matter lesions, which is the main pathogenic mechanism of VAD [3]. It has been proposed that accumulated myelin debris and local inflammation cascade secondary to cerebral chronic ischemia hinders endogenous white matter repair. Resident microglia/macrophage phagocytose myelin debris, which resolves excessive inflammation in the damaged white matter and promotes white matter recovery [4].

Pentoxifylline (PTX) is a non-specific phosphodiesterase inhibitor and a methylxanthine derivative which is extracted from cacao beans [5, 6]. PTX was approved by the National Formulary of China for patients with ischemic stroke in 2010. In addition to ischemic stroke, A few controlled clinical studies suggested that PTX could be a therapeutic option for VAD with the tendency to improve cognitive impairment [7–9], but there is no consensus on this point in the international community. Moreover, with the development of the research on cerebrovascular disease and dementia, the classification of vascular dementia is becoming more and more refined [1, 10]. There is still no effective treatment for vascular dementia caused by white matter injury associated with cerebral small vessel disease. Extensive research has shown that PTX could reduce the secretion of inflammatory cytokines such as tumor necrosis factor-α (TNF-α), interleukin-1β (IL-1β), IL-6, IL-8 by inhibiting microglia/macrophage activation in neuropathic pain and ischemic stroke models [11, 12]. Moreover, it was shown that PTX could regulate macrophage migration and myelin debris uptake in vitro [13]. However, the effect of PTX on microglial phagocytosis of myelin debris has not been confirmed.

Mer receptor tyrosine kinase (Mertk) belongs to TAM (Tyro3, Axl and Mertk) family, which is a professional phagocytic receptor expressed on the surface of microglia and other immune cells. All phagocytic processes start with the exposure of “eat-me” signal from the apoptotic cells or debris. For instance, phospholipid phosphatidylserine on apoptotic cells/myelin debris could act as a strong eat-me signal initiating microglia rapid recognition and clearance by Mertk [14, 15]. Efficient clearance of apoptotic cells by Mertk and Axl has fundamental roles facilitating adult neurogenesis [16]. In addition to apoptotic cell clearance, Mertk was required for myelin engulfment and subsequent remyelination in multiple sclerosis [17, 18].

A recently published study showed that activated peroxisome proliferator-activated receptors-γ (PPAR-γ) could augment the expression of Mertk in microglia and accelerate the clearance of hematoma after intracranial hemorrhage, suggesting that PPAR-γ may act as an upstream regulator of Mertk [19]. However, the protective patterns of Mertk and its transcriptional regulation in VAD model are yet to be examined.

Considering the above factors, we aimed to explore the therapeutic effects of PTX on cognitive function and the white matter integrity in mouse bilateral common carotid artery stenosis (BCAS) models. We hypothesized that PTX activated PPAR-γ and promoted white matter repair by modulating Mertk-mediated myelin clearance in microglia. These results could further enhance our understanding on PTX and provide a novel therapeutic strategy to limit white matter impairment after vascular dementia.

## Results

### PTX alleviated cognitive impairment after BCAS

In order to demonstrate whether PTX treatment alleviated cognitive impairment after BCAS, several behavioral tests were used. Dose effective screening of PTX is shown in Additional file 1: Fig. S1A–D.

**Fig. 1:**
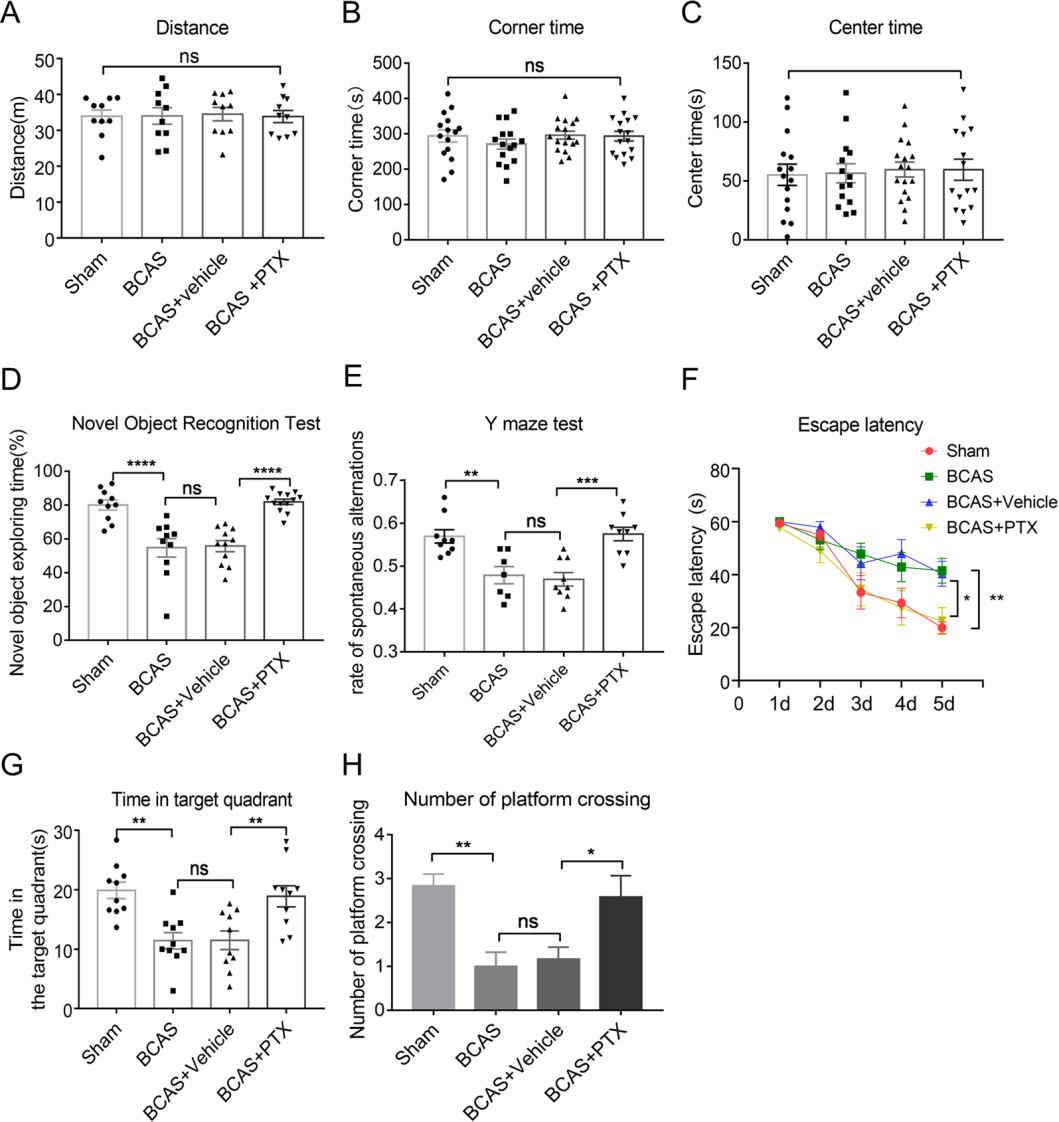
PTX alleviated cognitive impairment after BCAS. **A**–**C** The total distance moved (distance) (*n* = 10 mice per group), the percentage of time in the corner (corner time) (*n* ≥ 15 mice per group) and the percentage of time in the center (center time) (*n* ≥ 14 mice per group) of open field test. D, The exploratory preference to novel objects (*n* ≥ 10 mice per group) in the novel object recognition. **E** The percentage of spontaneous alternations in the Y-maze test (*n* ≥ 7 mice per group). **F**–**H** Escape latency during the acquisition phase (Days 1–5) (*n* ≥ 10 mice per group), the time in target quadrant (*n* = 10 mice per group) and the number of platform crossing (*n* = 12 mice per group) of the probe test (Day 6). All data were presented as the mean ± SEM. *p < 0.05, **p < 0.01, ***p < 0.005, **** < 0.001, “ns” means no significance (*p* > 0.05)

Open field test was applied to exclude the impact of locomotor activity and anxiety-like behavior on cognitive function. To be detailed, total distance reflected locomotor activity of mice. No significant differences were found in the total distance among the different treatment groups (Fig. 1A), indicating that the motor activity of mice was not affected by PTX or BCAS treatment. The corner time and center time were used to test anxiety-like behavior of mice. Depressed mice prefer to stay in the corners, therefore the central residence time decreases accordingly. There were no significant differences among all groups regarding displaying partialities towards corner areas or staying in the center of the field (Fig. 1B, C), indicating that the anxiety-like behavior was not affected by BCAS or PTX treatment.

Then, we observed whether PTX could impact characteristic exploratory feature of rodents by the novel object recognition test. The results showed that the BCAS group has less interest to explore novel object, as can be seen a notable reduction in exploratory preference time compared with sham group. However, PTX (50 mg/kg) treatment increased exploratory preference time compared with vehicle group. In the other words, PTX treatment could augment mouse exploratory tendency to novel object after BCAS (Fig. 1D). Next, we used Y-maze to evaluate the short-term working memory of mice. The results showed that the sham group exhibited a high rate of spontaneous alternations, whereas it was decreased in the BCAS group. However, the rate of spontaneous alternations was significantly increased by PTX (Fig. 1E).

At the same time, the Morris water maze test was used to determine the spatial learning and memory performance of mice. The quantitative data showed that at Day 1–5 in the training trials, the BCAS group has significantly longer escape latency compared with that of sham group (Fig. 1F). Likewise, BCAS group displayed a shorter time in target quadrant and smaller number of platform crossing of the probe trial (Day 6) (Fig. 1G, H); while PTX treatment gave a positive effect on spatial learning and memory, as evidenced by shorter escape latency, more numbers of platform crossing and longer time in target quadrant.

Taken together, using several different approaches, we found that cognitive impairment following BCAS could be alleviated by PTX administration.

### PTX reduced white matter damage after BCAS

**Fig. 2:**
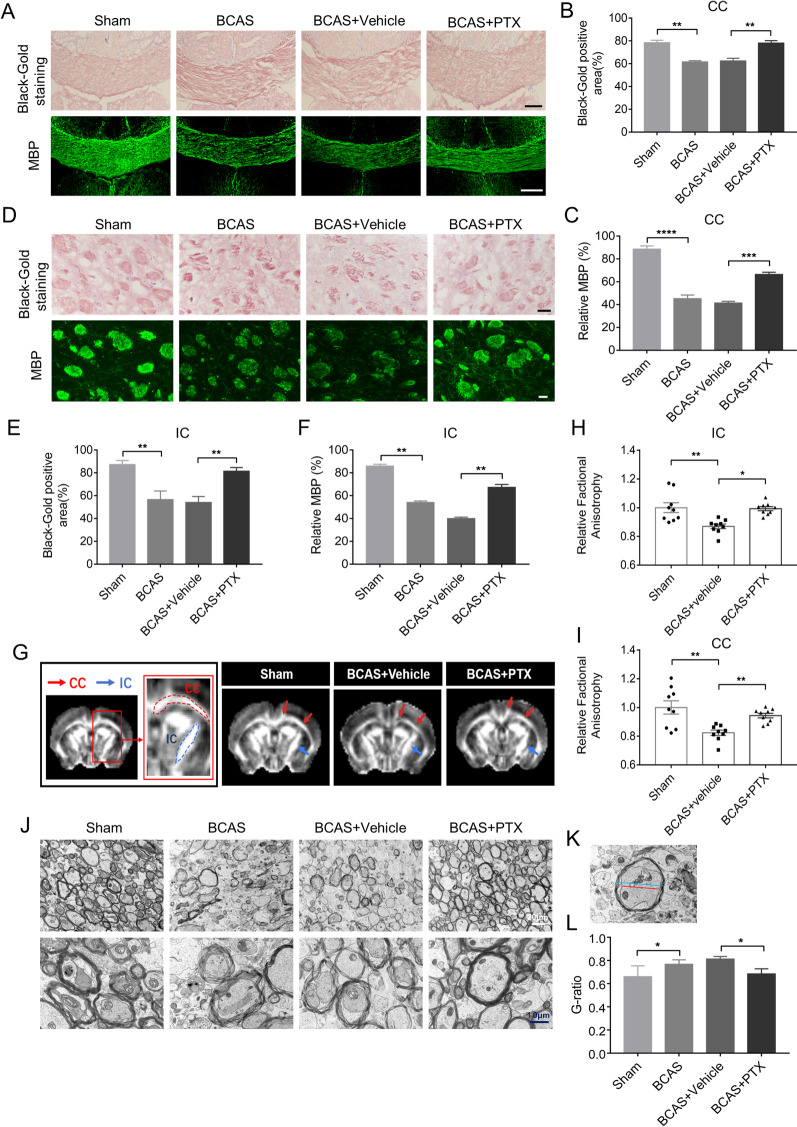
PTX alleviated white matter damage after BCAS. **A** Representative images of black-gold staining (lavender) and myelin basic protein (green) in corpus callosum (CC) at Day 30 after BCAS. Scale bar: 200 μm. **D** Representative images of black-gold staining (lavender) and myelin basic protein (green) in internal capsule (IC) at Day 30 after BCAS. Scale bar: 50 μm. **B**, **E** Quantification of immunofluorescent intensity of black-gold staining area in CC and IC (*n* ≥ 3 mice per group). **C**, **F** Quantification of immunofluorescent intensity of MBP area in CC and IC (*n* ≥ 3 mice per group). **G** Representative images of FA maps of mice brain; Red arrows indicate the CC, Blue arrows indicate the IC. H, Quantification of FA value in IC (*n* = 9 mice per group). **I** Quantification of FA value in CC (*n* = 9 mice per group). **J** Representative images of Electron microscopy in IC. **K** Schematic diagram showing the diameter of the axon (red line) and the diameter of the entire myelinated fiber (blue line). **L** Quantification of G-ratio (*n* ≥ 4 mice per group). All data were presented as the mean ± SEM. *p < 0.05, **p < 0.01, ***p < 0.005, ****p < 0.001

To evaluate the effect of PTX on white matter integrity after BCAS, we used MBP and black-gold staining. We found that PTX treatment increased MBP and black-gold staining intensity in the CC (Fig. 2A–C) and IC (Fig. 2D–F) area *versus* the vehicle group. Whole-brain MRI (Fig. 2G–I) were acquired at Day 30 after BCAS. Fractional anisotropy (FA) map showed higher FA value (IC: 0.8236 ± 0.01847 in vehicle group vs*.* 0.9435 ± 0.01582 in PTX group, *p* = 0.0252; CC: 0.8701 ± 0.01614 in vehicle group vs*.* 0.9927 ± 0.01397 in PTX group, *p* = 0.0029) in PTX group than vehicle-treated group (Fig. 2H, I). Furthermore, electron microscopy (Fig. 2J–L) was performed at Day 30 after BCAS and PTX group demonstrated decreased G-ratio (0.8119 ± 0.01168 in vehicle group vs*.* 0.6842 ± 0.02006 in PTX group, *p* = 0.0322) (Fig. 2L) relative to the vehicle group. These results indicated that PTX treatment preserved white matter integrity in BCAS mice.

### PTX upregulated Mertk expression and promoted microglia phagocytosis after BCAS

**Fig. 3:**
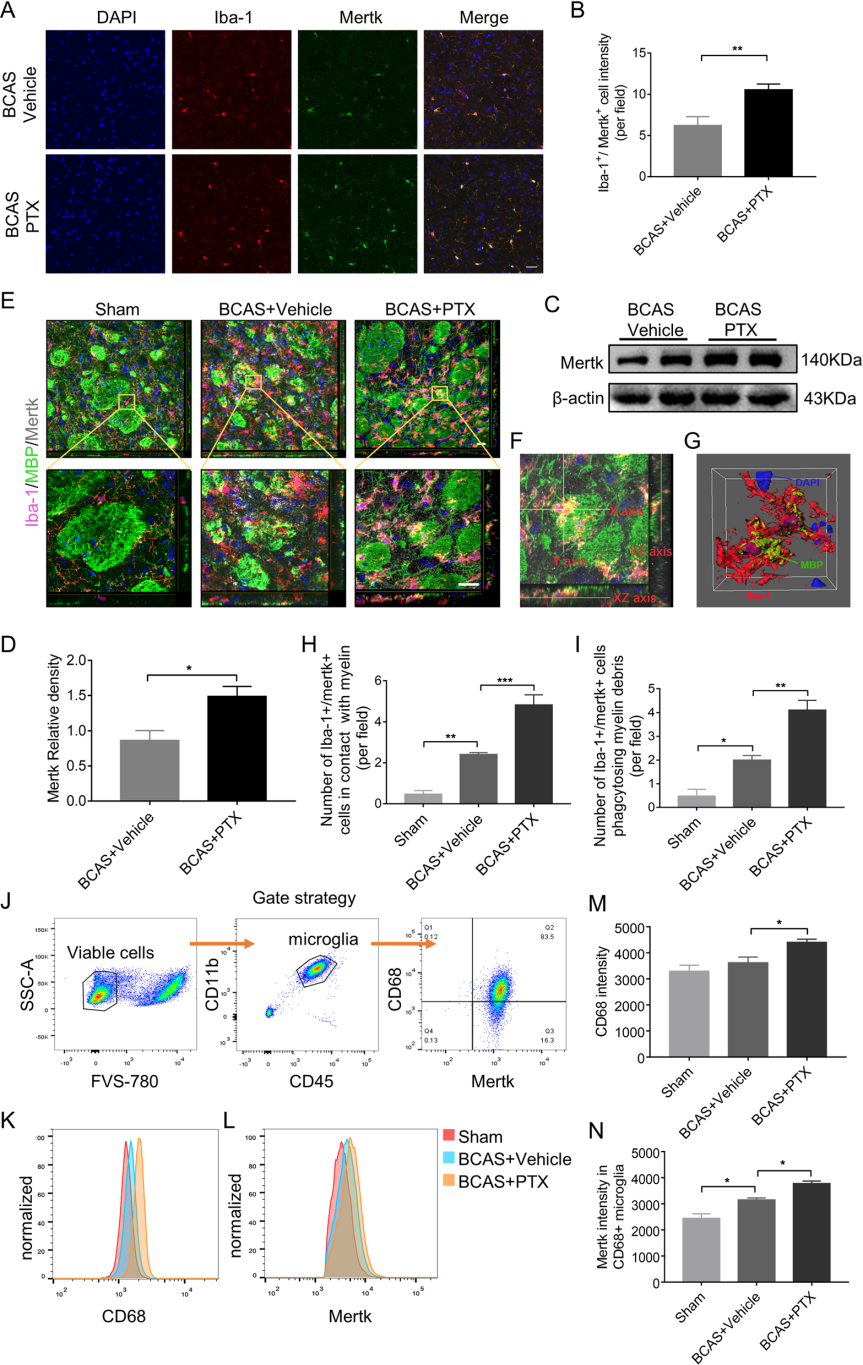
PTX promoted microglia phagocytosis and upregulated Mertk expression after BCAS. **A** Immunofluorescent images of Iba-1 (red)/Mertk (green)/DAPI (blue) colocalization in IC at Day 30 after BCAS. Scale bar: 20 μm. **B** Quantification of Iba-1 and Mertk double-positive cells (*n* = 4 mice per group). **C** Representative immunoblots probed with antibodies against Mertk and β-actin at Day 30 after BCAS. **D** Quantification of the levels of Mertk normalized to β-actin (*n* = 3 mice per group). **E** Immunostaining of Iba-1 (red)/ MBP (green)/Mertk (grey)/DAPI (blue) in IC at Day 30 after BCAS. **F**, **G** 3D-confocal images of Iba-1 (red) phagocytosing MBP debris (green). Scale bar: 40 μm. **H** Quantification of Iba-1 and Mertk double-positive cells adhered to MBP (*n* = 3 mice per group). **I** Quantification of Iba-1 and Mertk double-positive cells phagocytosing MBP (*n* = 3 mice per group). **J** Gate strategy for microglia isolation at the Day 30 after BCAS via FACS. **K**, **M** CD68 expression intensity of isolated microglia in each group (*n* = 4 mice per group). Detailed gating strategy can be found in the Additional file 3: Fig. S3C. **I**, **N** Mertk expression intensity in CD68 + microglia in each group (*n* = 4 mice per group). All data were presented as the mean ± SEM. *p < 0.05, **p < 0.01, ***p < 0.005

We detected Mertk expression of microglia by double-labeling Iba-1 and Mertk in mouse BCAS slices. As shown in Fig. 3A, B, the number of Iba-1^+^Mertk^+^ microglia in the IC was increased in PTX-treatment group compared with the vehicle group (6.223 ± 0.619 per field in vehicle group vs*.* 10.55 ± 0.3998 per field in PTX group, *p* = 0.0042). Consistently, Mertk protein level was significantly increased in IC after PTX treatment via western blotting (Fig. 3C, D). Subsequently, we detected phagocytosis of Mertk-positive microglia. In IC distinct of BCAS model, damaged myelin bundles were surrounded by Mertk-positive microglia. Quantitatively, PTX treatment enhanced the spatial contact between damaged myelin bundles and Mertk-positive microglia compared with the vehicle group (Fig. 3E, F, H; Additional file 1: Fig. S1E, F). By 3D-confocal, we confirmed that the microglia around myelin bundles could phagocytose myelin debris (Fig. 3G). PTX treatment promoted microglial phagocytosis of myelin debris compared with the vehicle group (Fig. 3I). Then, we sorted microglia (CD45^int^CD11b^+^) and characterized its phagocytic activity by evaluating CD68 and Mertk intensity. Our data demonstrated that PTX injection enhanced CD68 intensity in microglia population (3624 ± 214.8 in vehicle group vs. 4405 ± 121.8 in PTX group, *p* = 0.0485) (Fig. 3K, M). Additionally, in CD68-positive microglia population, Mertk intensity increased in PTX group (Fig. 3L, N), indicating microglia-mediated engulfment in BCAS could be enhanced by PTX. Moreover, we detected various inflammatory factors in IC by RT-PCR and found that BCAS group increased the mRNA level of TNF-α compared with the sham group, yet PTX could lower TNF-α at the transcriptional level (Additional file 2: Fig. S2A).

In summary, PTX increased the expression of the phagocytic receptor Mertk on the surface of microglia and promoted its phagocytosis after BCAS. At the same time, it reduced the release of inflammatory cytokines of TNF-α induced by BCAS.

### PTX upregulated Mertk expression and promoted phagocytosis in primary microglia exposed to myelin debris

**Fig. 4:**
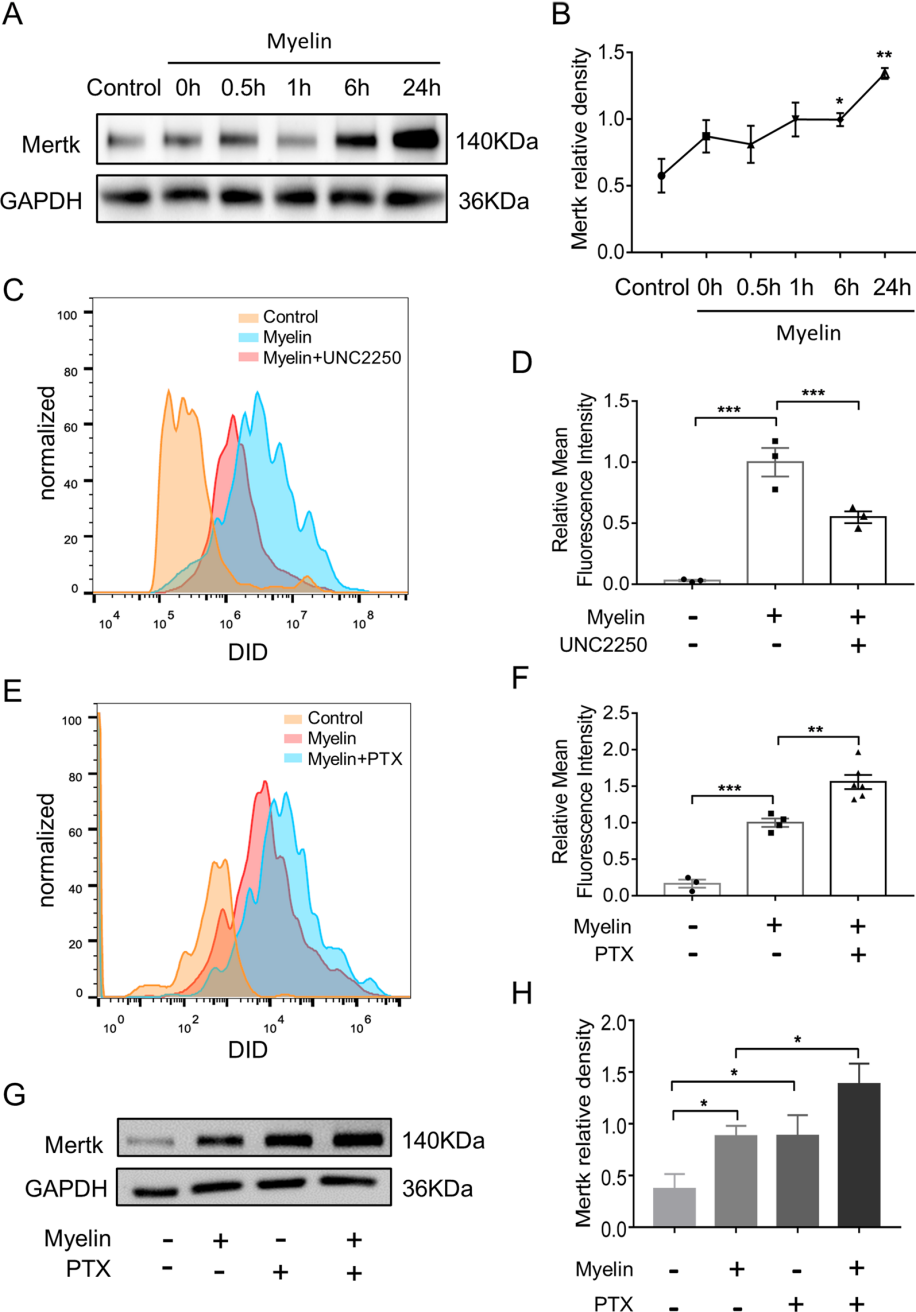
PTX upregulated Mertk expression and promoted phagocytosis in primary microglia exposed to myelin debris. **A** Representative immunoblots probed with antibodies against Mertk and GAPDH at different time points after myelin stimulation. **B** Quantification of Mertk bands normalized to GAPDH (*n* = 3 repeats per group). **C**, **D** Primary microglia were treated with UNC2250 (100 nM) for 2 h, and then con-cultured with myelin debris (0.01 mg/ml) stained with DID for 0.5 h, primary microglia were collected and myelin debris signal intensity in microglia were conduct by FACS (*n* = 3 repeats per group). **E**, **F** Primary microglia were treated with vehicle or PTX (25 μM) for 2 h, and then incubated with myelin debris (0.01 mg/ml) for 0.5 h. Myelin debris signal intensity in microglia were detected by FACS (*n* ≥ 3 repeats per group). **G** Representative immunoblots probed with antibodies against Mertk and GAPDH. **H** Quantification of the levels of Mertk normalized to GAPDH (*n* = 3 repeats per group). All data were presented as the mean ± SEM. *p < 0.05, **p < 0.01, ****p < 0.001

We first examined the protein level of Mertk in myelin-activated microglia and found Mertk was elevated gradually within 24 h after myelin stimulation (Fig. 4A, B). Then, we applied Mertk inhibitor UNC2250 (100 nM) to myelin-activated microglia. We detected the fluorescence intensity of FITC^+^ myelin debris in APC^+^ microglia to represent the capability of myelin debris engulfed by microglia (Fig. 4C). The result showed that UNC2250 could inhibit microglial phagocytosis of myelin debris (Fig. 4D). Furthermore, microglia were co-cultured with the vehicle or PTX for 2 h and then exposed to myelin debris for 0.5 h. We found that PTX treatment improved the ability of microglia to phagocytose myelin debris (1 ± 0.05644 in vehicle group vs. 1.559 ± 0.0967 in PTX group, *p* = 0.0025) (Fig. 4E, F). In addition, using western blotting, we detected Mertk expression in primary microglia treated by PTX (Fig. 4G) and found that Mertk protein level was significantly increased in microglia treated by PTX (Fig. 4H). In summary, PTX upregulated Mertk expression and promoted phagocytosis of myelin debris in primary microglia.

### PTX upregulated Mertk by stimulating PPAR-γ nuclear translocation in microglia after BCAS

**Fig. 5:**
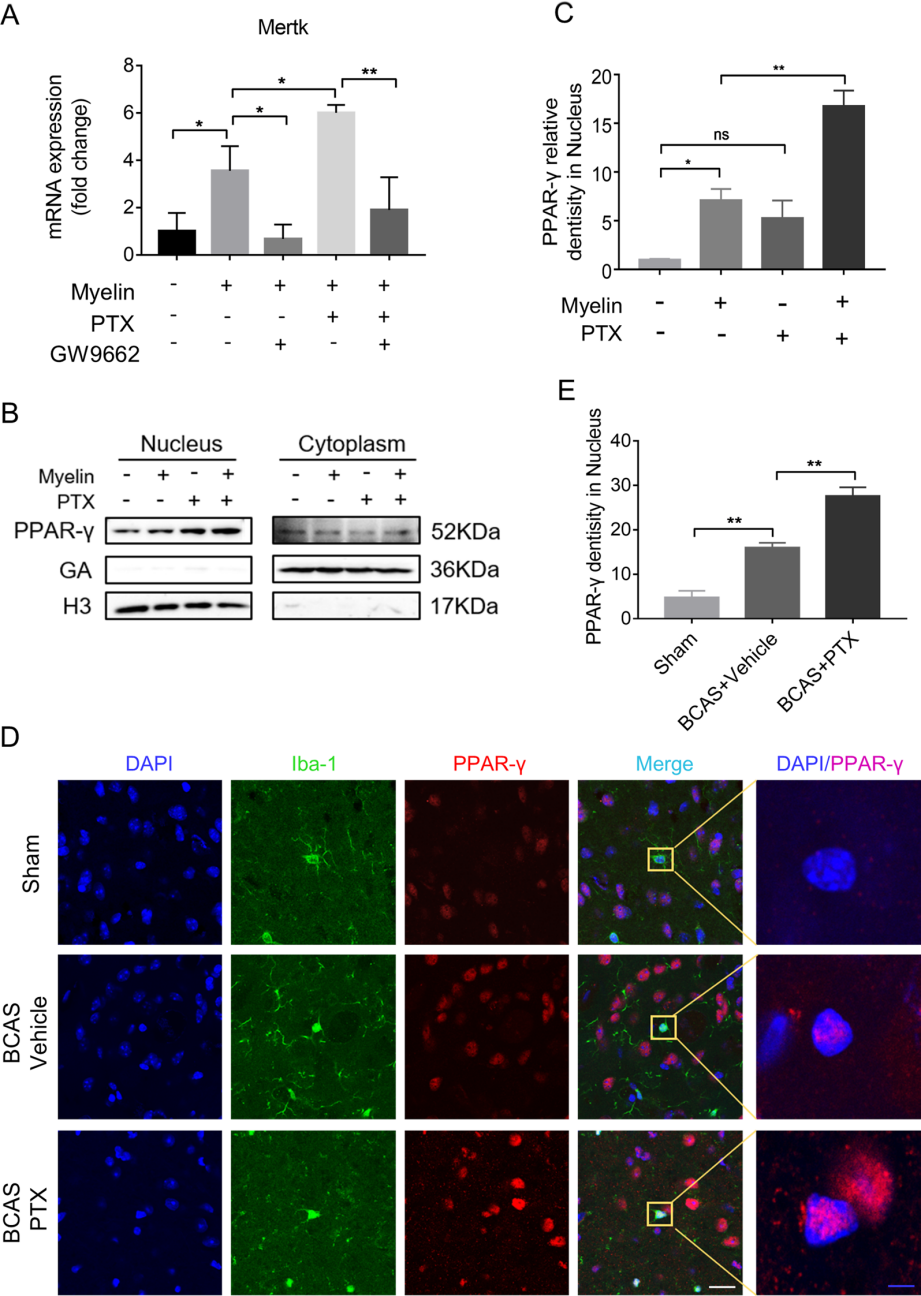
PTX upregulated Mertk by stimulating PPAR-γ nuclear translocation in vivo and in vitro. **A** Primary microglia were incubated with GW9662 (10 μM) for 1 h, followed with myelin debris (0.01 mg/ml) with or without PTX (25 μM) for 6 h. Quantitative RT-PCR analysis of Mertk mRNA in primary microglia (*n* = 3 repeats per group). **B** Representative immunoblots probed with antibodies against PPAR-γ, GADPH and H3 in nucleus and in cytoplasm of BV2 cells. **C**, Quantification of PPAR-γ levels normalized to H3 in nucleus (*n* = 3 repeats per group). **D** Immunofluorescent images of Iba-1 (green)/PPAR-γ (red)/DAPI (blue) colocalization in IC at Day 30 after BCAS. White scale bar: 20 μm, blue scale bar: 4 μm. E, Quantification of immunofluorescent intensity of PPAR-γ in DAPI area. The values were normalized to those of the control group (*n* = 4 mice per group). All data were presented as the mean ± SEM. *p < 0.05, **p < 0.01, “ns” means no significance (*p* > 0.05)

To further explore how PTX increased the expression of Mertk, we applied PPAR-**γ** inhibitor GW9962. Primary microglia were incubated with myelin (0.01 mg/ml), myelin (0.01 mg/ml) + GW9962 (10 μM), myelin (0.01 mg/ml) + PTX (25 μM), myelin (0.01 mg/ml) + PTX (25 μM) + GW9962 (10 μM) for 6 h. Then, we detected the transcriptional levels of Mertk by RT-PCR. The results demonstrated that Mertk upregulation by PTX could be reversed by GW9962 (Fig. 5A), indicating that PTX increased Mertk probably through activating PPAR-γ pathway. To further confirm this point, we extracted nuclear and cytoplasmic protein separately to detect nuclear translocation of PPAR-γ in BV2 cells and primary microglia by western blotting (Fig. 5B; Additional file 2: Fig. S2B). Upon myelin or PTX stimulation, PPAR-γ accumulated profoundly in the nuclear, especially in the myelin + PTX group (Fig. 5B, C). In addition, we detected PPAR-γ expression pattern in mouse BCAS slices by double-labeling Iba-1 and PPAR-γ. As shown in Fig. 5D, E, BCAS mice receiving PTX presented pronounced PPAR-γ nuclear translocation compared to vehicle group in microglia (16.01 ± 1.065 in vehicle group vs. 27.65 ± 1.926 in PTX group, *p* = 0.0012). Similarly, PTX could promote PPAR-γ nuclear translocation in primary microglia (Additional file 2: Fig. S2C, D). Collectively, our data demonstrated that PTX could promoted PPAR-γ nuclear translation in vivo and in vitro.

### PTX improved cognitive impairment and white matter damage dependent on Mertk

**Fig. 6:**
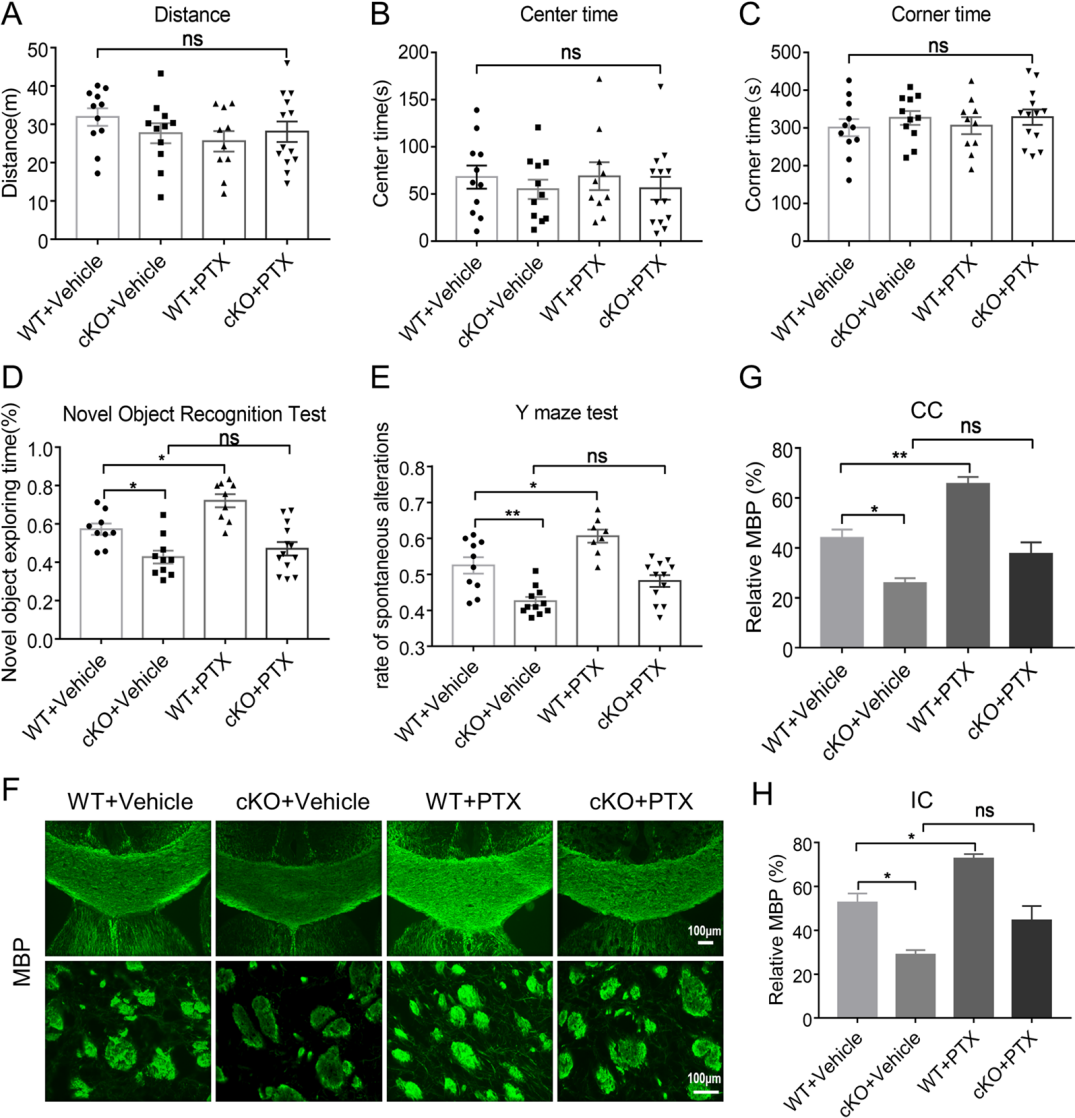
PTX improved cognitive impairment and white matter integrity dependent on Mertk. The total moved distance (**A**), the percentage of time in the corner (**B**) and the percentage of time in the center (**C**) (*n* ≥ 10 mice per group) detected by open field test. **D** The exploratory preference to novel objects (*n* ≥ 9 mice per group) in the novel object recognition. **E** The percentage of spontaneous alternations in the Y-maze test (*n* ≥ 8 mice per group). **F** Representative images of myelin basic protein (green) in corpus callosum (CC) and Internal Capsule (IC) at Day 30 after BCAS. Scale bar: 100 μm. **G**, **H** Quantification of immunofluorescent intensity of MBP area in CC and IC (*n* = 3 mice per group). All data were presented as the mean ± SEM. *p < 0.05, **p < 0.01, “ns” means no significance (*p* > 0.05)

To test whether Mertk plays a fundamental role in PTX mediated white matter protection, we applied microglia-specific Mertk knockout mice and wild-type (WT) mice. Mertk was negative in Iba-1 + microglia in conditional knockout (cKO) mice, while positive in wild-type littermate, indicating that Mertk has been specifically knocked out in microglia of the cKO mice (Additional file 2: Fig. S2E). No significant differences were found in the behavior tests between cKO mice and wild-type littermate (Additional file 2: Fig. S2F–K). We categorized the animals into four groups: WT + BCAS, Mertk cKO + BCAS, WT + BCAS + Vehicle, Mertk cKO + BCAS + PTX. Firstly, we detected open field test and found no significant difference about total distance traveled, residence time in the center among all groups (Fig. 6A–C). Novel object recognition test showed that the cKO group has less interests to explore novel object compared to the WT group, and PTX failed to elevate it (Fig. 6D). Furthermore, Y-maze showed that the cKO group exhibited a lower rate of spontaneous alternations than WT group, and it was not increased after the treatment of PTX (Fig. 6E). These behavior data suggested that Mertk cKO exacerbated cognitive function of BCAS, while PTX mediated protection was dependent on Mertk expression. In addition, we used MBP staining (Fig. 6F) to detect white matter integrity in each group. Consistent with the behavior test, Mertk cKO group demonstrated less MBP intensity in the CC and IC areas compared with its wild littermates. Although we still observed the protection of PTX in WT groups, PTX was ineffective at reducing white matter injury in Mertk cKO mice (Fig. 6G, H). In summary, conditional knockout of Mertk in microglia aggravated white matter damage and cognitive dysfunction, whereas could not reversed by PTX after BCAS, suggesting the importance of Mertk in BCAS and the protective action of PTX.

## Discussion

White matter injury caused by hypoperfusion or hypoxia participated in the development of vascular dementia. BCAS mouse model is widely accepted to mimic the cognitive impairment and white matter injury of clinical vascular dementia [20–22]. In this study, we demonstrated that short-term working memory, characteristic exploratory feature, spatial learning and memory performance of mice were disrupted following BCAS, whereas this cognitive impairment was reversed by the administration of PTX. It is noteworthy that locomotor activity and behavioral despair could be affected in severe BCAS model. In such cases, the outcomes of the MWM cannot be attributed only to cognitive function [23, 24]. Therefore, we performed open field test to exclude the impact of the locomotor activity and behavioral despair. After BCAS, white matter lesions were often observed in the corpus callosum, caudate putamen, internal capsule, and to a lesser extent in the optic tract [25]. Consistently, our data also showed that myelin and axon integrity were damaged in CC and IC area 30 days after BCAS. With the application of MRI, immunofluorescence and electron-microscopy, we strongly confirmed that PTX increased white matter integrity after BCAS. Clinical trials suggested a trend toward improved cognitive function in VAD patients administrated with pentoxifylline [26]. To our knowledge, this is the first study confirming its efficacy in VAD animal model with detailed behavior, imaging and pathological data. Using animal model of maternal LPS-induced white matter injury, Mustafa et al. demonstrated that PTX may minimize periventricular leukomalacia in the developing rat brain [27]. This is the only evidence showing the protective potency of PTX in white matter injury. Moreover, mechanisms responsible for the neuroprotective actions of PTX were largely unknown.

Myelin debris is a major obstacle to white matter repair both as a physical impediment [28] and through expressing axon growth inhibitory signals [29]. Microglia could be highly efficient in clearing myelin debris from demyelination which is critical for oligodendrocyte precursor cell recruitment and their maturation into oligodendrocytes [30]. Modulating microglia-mediated myelin debris-clearance could be a therapeutic target to improve white matter injury [31]. In Wallerian degeneration, PTX may regulate different effector functions of macrophages such as migration and myelin phagocytosis [32]. Therefore, we hypothesis that PTX might promote myelin debris scavenge by microglia. In this study, we found that microglia in PTX group localized around damaged white matter bundles, showing higher capacity of phagocytosing myelin debris after BCAS.

The phagocytic receptor Mertk belongs to TAM receptors, which sense eat-me signals (for instance, phosphatidylserine of lipid-rich myelin debris) from the extracellular space and initiates debris clearance [33]. The functional roles of Mertk in white matter injury have been addressed in multiple sclerosis (MS) [18, 34]. Peripheral blood monocyte-derived macrophages of MS patients displayed a decreased Mertk expression and reduced ability to phagocytose human myelin [18, 34]; Likewise, Mertk knockout mice showed impaired clearance of myelin debris and remyelination in cuprizone-mediated demyelination [17]. In addition to central nervous system (CNS), Mertk expressed on Schwann cells is critical for myelin clearance in a mouse model of peripheral nerve crush injury [35]. Generally, no studies concerned functional profile of Mertk in the pathogenesis of ischemic white matter injury. In this present study, microglia-specific Mertk knockout mice demonstrated equal memory compared with their wild littermates in the intact status. After BCAS, Mertk knockout mice showed cognitive decline and white matter damage much more pronounced than their age matched controls, indicating that Mertk gene is related to an increased susceptibility of ischemic white matter injury.

Interestingly, in this study, PTX treatment upregulated Mertk expression in microglia after BCAS and in myelin debris-stimulated microglia. This regulation is dependent on the activation of PPAR-γ, since PPAR-γ antagonist could abolish Mertk upregulation by PTX. PPAR-γ is a type of ligand-activated transcription factor under the class of nuclear receptor superfamily [36]. Studies have demonstrated that PPAR-γ activation could reduce pro-inflammatory cytokines such as TNF-α and promote white matter integrity after ischemic stroke [37, 38]. In mouse models of intracerebral hemorrhage, activated PPAR-γ augmented the expression of Mertk and accelerated the phagocytosis of microglia and the clearance of hematoma [19]. PTX is a phosphodiesterase inhibitor that modulates inflammatory response through increasing intracellular cyclic adenosine monophosphate (AMP) concentration and reducing TNF-α gene transcription [26]. In this study, we found that PTX treatment facilitated PPAR-γ translocation into the nucleus after BCAS in mice and in primary cultured microglia, which is a critical for myelin clearance.

There are several limitations to the present study. BCAS model has some drawbacks. Firstly, BCAS model could not reflect actual small vessel dysfunction of cerebral small vessel disease (CSVD) in clinic. Secondly, other vascular risk factors that induce white matter lesions, such as hypertension, diabetes, aging and gender could not be reproduced by the BCAS model [21]. Thirdly, although BCAS model could mimic chronic hypoperfusion, which is the main pathogenic mechanism of white matter lesion and vascular cognitive impairment, it is not identical to the pathogenetic mechanism of the patient. Cerebral blood flow in BCAS model decreased dramatically within the first postoperative day and then slowly gradually returned to a more stable hypoperfusion level [39], but cerebral hypoperfusion in patients with vascular dementia progressed slowly and lasted for a long time. Thus, in the future, novel and more reliable animal models are needed to study the pathogenesis of vascular dementia as well as the mechanism of PTX. Moreover, BCAS lacks an in vitro model, and we used myelin debris stimulation to mimic the demyelination process in vivo, which could not reflect the complicated pathogenesis observed in vivo. In summary, we demonstrated that PTX may represent an effective therapeutic strategy for patients with VAD, via promoting microglial phagocytosis of myelin debris depending on Mertk.

## Conclusions

PTX ameliorated cognitive impairment and white matter damage induced by BCAS. PTX treatment increased the expression of Mertk and microglia-mediated myelin clearance via activating PPAR-γ. Moreover, these protective effects of PTX were abolished in microglial conditional Mertk knockout mice. Additionally, PTX prominently restrained the expression of inflammatory cytokine TNF-α. Therefore, our work here addressed a novel role of PTX in modulating microglia-associated phagocytosis and protecting against white matter injury after BCAS, which provided more evidences for clinal applications of PTX in VAD patients.

## Materials and methods

### Mice

Male C57BL/6J mice weighing 25–28 g were provided by the Animal Center of Nanjing Drum Tower Hospital. Mice were randomly divided into four groups: the sham-operated (sham) group; BCAS group; the saline-treated BCAS (BCAS + Vehicle) group; and the 50 mg/kg PTX-treated BCAS (BCAS + PTX) group. Microglia-specific Mertk knockout mice (Cx3cr1-Cre: Mertk^fl/fl^) and wild-type littermate (Mertk^fl/fl^) were purchased from the GemPharmatech Co., Ltd. (Nanjing, Jiangsu, China). The mice that met both Mertk flox homozygosity and Cx3cr1-Cre heterozygosity were the microglia-specific Mertk knockout mice (Cx3cr1-Cre: Mertk^fl/fl^) (Additional file 3: Fig. S3A, B). All animal experimental protocols were approved by the Standard Medical Laboratory Animals Care. Microglia-specific Mertk knockout mice and wild-type littermate were simultaneously randomly assigned to two groups: the saline-treated BCAS (BCAS + Vehicle) and the 50 mg/kg PTX-treated BCAS (BCAS + PTX) group.

### BCAS surgery

The BCAS model was performed according to previous experiments [25]. Briefly, mice were anaesthetized with 4% chloral hydrate (10 ml/kg) by intraperitoneal injection. After exposing both common carotid arteries (CCAs) through a midline cervical incision, a micro-coil (Inner diameter 0.18 mm, pitch 0.50 mm, total length 2.5 mm, purchased from Sawane Spring Co.) was rotated around the right CCA in a twining way. After 30 min, another micro-coil of the same size was twined around the left CCA.

### Cell culture

Primary microglia were isolated and purified from 1-day-old C57BL/ 6J mice as described previously [21]. In brief, brain membrane was removed and cerebral cortex was digested with 0.25% tripsin EDTA for 10 min. Then, we added the same amount of DMEM medium containing 10% FBS to terminate the digestion. The cells were centrifuged at 800 rpm at 37 ℃ for 10 min. The supernatant was aspirated and cells were cultured in 75 cm^2^ flasks for 10–12 days, and then the microglia were separated from mixed primary glia by shaking the flasks and the floating microglia were replanted onto plates for about 48 h. The primary microglia were cultured in 90% DMEM (Invitrogen, Frederick, MD, USA), 10% fetal bovine serum (FBS, Hyclone, Logan, UT, USA) at 37 ℃ in a humidified atmosphere of 5% CO_2_. The murine microglia cell line BV2 was prepared from the China Infrastructure of Cell Line Resources (Beijing, China) and its culture medium was the same as primary microglia.

### Myelin debris preparation and stimulation

Mice were euthanized according to CO_2_ asphyxiation guidelines, followed by cervical dislocation. Brains were isolated in 0.32 M sucrose solution and cut into pieces approximately 5 × 5 × 5 mm^3^. 90 ml homogenized brain solution were gently added the top of the 0.83 M sucrose solution. After centrifuging at 100,000 rpm for 45 min at 4 °C, the crude myelin debris were collected from the interface of the two sucrose densities, dissolved in 35 ml using Tris·Cl buffer solution and homogenized for 30–60 s. After centrifuging at 100,000 rpm for 45 min at 4 °C, the pellets were resuspended in 10–15 ml of Tris·Cl buffer solution and then centrifuged at 100,000 rpm for 45 min. The pellets were resuspended in 5–6 ml of sterile phosphate buffer solution (PBS) and centrifuged at 22,000 rpm for 10 min t 4 °C. The pellets were myelin debris we need. Finally, we resuspend the pellets in PBS to a final concentration of 100 mg/ml for future use. We used 0.01 mg/ml myelin debris to stimulate primary microglia for different time and detected the protein level of Mertk. Moreover, pre-treated with Mertk inhibitor UNC2250 (100 nM), primary microglia were incubated with myelin debris (0.01 mg/ml) for 6 h and then used for flow cytometry. Finally, primary microglia were incubated with myelin (0.01 mg/ml), myelin (0.01 mg/ml) + GW9962 (10 μM), myelin(0.01 mg/ml) + PTX (25 μM), myelin(0.01 mg/ml) + PTX(25 μM) + GW9962 (10 μM) for 6 h.

### Open field test

To determine motor function and anxiety-like behavior, a 50 × 50 × 50cm^3^ box was placed in a room with sound insulation, appropriate light intensity, temperature and humidity. The bottom of the box is divided into 16 small squares. The recording started immediately after the mouse is placed in the center of the box and continued for ten minutes. Total distance traveled and time spent in the center area and corner area were measured and recorded by ANY-maze software (Stoelting, USA).

### Novel object recognition test

In the adaptive trail, the mouse was placed in a box without any objects for 15 min on three consecutive days. Twenty-four hours after the last adaption, the training trail started. Two identical objects were placed in the box, and the mice were allowed to freely explore in the box for 10 min. An hour later, one randomly selected familiar object was replaced with a novel one, and the mouse was left to explore for another five minutes. The olfactory trace was removed by 70% alcohol after each trial. Climbing and sitting on an object is not designated as an exploration, but sniffing with the nose or touching with the forepaws is designated as an exploration. The percentage of exploratory preference is as follows: time to explore a novel object/total time to explore both two objects.

### Y-maze test

Y-maze test was used to test short-term working memory as previously described [40]. The Y-maze made of dark polyvinyl plastic consists of three arms (marked as A, B, C). Mice were individually placed on the junction of the arms and move freely over an 8-min period. Spontaneous alternations were manually recorded when mice consecutive entered into all three different arms (i.e., ABC, ACB, CAB, BCA, CBA, and BAC). The percentage of spontaneous alternations (%) was defined as the number of spontaneous alternations in behavior/(the total number of arm entries − 2) × 100.

### Morris water maze test

Morris water maze test was performed to evaluate spatial learning and memory in the mice. The apparatus is a 1.6-m-diameter pool surrounded by four curtains. The pool was divided equally into four quadrants, with a hidden platform in the fourth quadrant. Before the experiment, the pool was filled with water 1–2 cm above the platform. During the acquisition phase (Day 1–5), the mice were trained once a day and placed in four different quadrants to search for a platform for one minute at a time. A period of searching time is known as latency time. Mice that have not found the platform in 1 min will be guided by the experimenter to stay on the platform for 30 s. In the probe test (Day 6), the platform was removed and a 60-s exploration experiment was performed to investigate the memory of the mouse on the platform. Latency time, the number of platform crossing and time in target quadrant were recorded by ANY-maze software (Stoelting, USA) automatically.

### Western blot analysis

The cells and tissues were homogenized in RIPA buffer containing protease inhibitor. Protein concentrations were determined by the BCA Assay (Thermo Fisher Scientific), equal amounts of which were loaded on gels and transferred to PVDF membrane (EMD Millipore). Membranes blocked with 5% milks were probed with the indicated primary antibodies: Mertk (Abcam, ab95925, 1:1000), PPAR-γ (Abcam, ab45036, 1:500), Histone H3 (Cell Signaling Technology, 4499S, 1:2000), GAPDH (BioWorld, AP0066, 1:5000).

### Real-time PCR

Total RNA was extracted from the brains and cells using the TRIzol reagent (Invitrogen) according to the manufacturer’s protocol. Then cDNA was reverse transcribed with a PrimeScript RT Reagent Kit (Takara). Finally, ABI StepOne Plus PCR instrument (Applied Biosystems) with a SYBR green kit (Applied Biosystems) was used to perform quantitative PCR. The expression of target gene levels was normalized to the endogenous control GADPH. The primer sequences were as follows:

**Table:** 

TNF-α	Forward	AGTCTGCACAGTTCCCCAAC
	Reverse	TTAGGAAGACACGGGTTCCA)
Mertk	Forward	AAGGTCCCCGTCTGTCCTAA
	Reverse	GCGGGGAGGGGATTACTTTG
GAPDH	Forward	GCCAAGGCTGTGGGCAAGGT
	Reverse	TCTCCAGGCGGCACGTCAGA

### Immunofluorescence staining

Briefly, 20-μm-thick coronal sections was fixed with 4% paraformaldehyde and then blocked with 5% BSA and 0.25% Triton X-100. Subsequently, brain sections were incubated with primary antibodies overnight at 4 °C. The primary antibodies were as follows: Rat anti-MBP (Abcam, ab7349, 1:500), Rabbit anti-Iba-1 (Abcam, ab178847, 1:500), Goat-anti-Mertk (R&D systems, AF591, 1:300), Rabbit anti-PPAR-γ (Abcam, ab45036, 1:500). After incubating with appropriate secondary antibodies (Invitrogen) at room temperature for 2 h, sections were observed by a fluorescence microscope (Olympus IX73) or confocal laser-scanning microscope (Olympus FV3000).

### Black-gold staining

After preheating 0.3% black-gold and 1% sodium thiosulfate solution to 60 °C, the sections were incubated in black-gold solution at 60 °C for about 12 min until the finest myelin fibers were stained dark red. Then the sections were incubated in sodium thiosulfate solution at 60 ℃ for 3 min. The sections were incubated in cresyl violet solution for 3 min at room temperature. Finally, after alcohol gradient dehydration, xylene clearing for 2 min, and resin mounting, the images were screened by BX Series.

### Electron microscopy

After the mice were euthanized, a 1 × 1 × 1 mm^3^ piece of tissue from the corpus callosum was taken and fixed in 2.5% glutaraldehyde. The following processing procedure was performed as previously reported protocol [41]. In brief, the blocks of tissue were incubated in 2% paraformaldehyde in PBS at 4℃ overnight and then fixed by 2% osmium tetroxide in PBS. After alcohol gradient dehydration, samples were embedded in epoxy resin. Subsequently, semi-thin sections which were cut with an ultramicrotome were stained with 1% toluidine blue, uranyl acetate and lead citrate. Then, the images were screened by transmission electron microscopy (Hitachi, HT7800). G-ratio, which is referred to the diameter of the axon/the diameter of the entire myelinated fiber, is used for the indicator.

### MRI

Magnetic resonance images were acquired on a 9.4T Bruker MR system (BioSpec 94/20 USR, Bruker) using a 440-mT/m gradient set with an 86-mm volume transit RF coil and a single channel surface head coil. Mice were anesthetized using isoflurane inhalation (2.5–3%) and monitored to maintain constant physiological parameters. Tooth bar and ear bars were used to restrain mice on a mouse holder for data acquisition. T2-weighted images were acquired using the 2D RARE (rapid acquisition with relaxation enhancement) sequence with the following parameters: repetition time (TR): 2500 ms, echo time (TE): 33 ms, field of view (FOV): 20 mm × 20 mm, matrix: 256 × 256 and 22 adjacent slices of 0.7 mm slice thickness. Diffusion-weighted images were acquired with spin-echo echo-planar imaging (SE-EPI) sequence with the following parameters: Two *b*-values (*b* = 0 and 1000 s/mm^2^) along with 30 non-collinear directions, δ = 4.1 ms, Δ = 10.3 ms; TR: 1500 ms, TE: 23.27 ms, FOV: 20 mm × 20 mm, matrix: 128 × 128, and 22 adjacent slices of 0.7 mm slice thickness. Images were converted into NIFTI format using MRIcron. Diffusion data were post-processing using FSL (v.5.0.9) pipeline including corrections for eddy currents and movement artifacts (*eddy_correct*), rotations of gradient directions according to eddy currents corrections (*fdt_rotate_bvecs*), brain mask extractions based on b0 images (*bet*) and FA maps calculations by fitting a diffusion tensor model at each voxel (*dtifit*). EC and IC were drawn using the itk-SNAP to extract the FA values.

### Flow cytometry and microglia isolation

Mice were trans-cardial perfused with 50 ml cold PBS containing 5 IU/ml heparin. Ischemic brains were isolated in 1 × HBSS with 25% glucose and HEPES. After thorough grinding, the total mixture passed through a 70-μm pore filter. Harvested single-cell suspensions were stratified on a 30–70% Percoll gradient (GE Healthcare BioSciences). After centrifuging at 2500 rpm at slow acceleration and deceleration for 20 min, cells at the interface were collected and stained with anti-mouse CD45 (BioLegend, 103114, 1:500), CD11b (Invitrogen, 11-0112-82, 1:500), CD68 (BioLegend, 137007, 1:300), Mertk (Invitrogen, 12-5751-82, 1:500). Among viable cells (FVS-780^low^), microglia (CD45^int^CD11b^+^) were isolated and analyzed by Fluorescence activated cell sorter (FACS) (BD Biosciences, Carlsbad, CA, USA).

### Statistical analysis

Statistical analysis was performed using SPSS 18.0 software (IBM Corp, Armonk, NY, USA) and data were expressed as the mean ± standard error of the mean (SEM). The normality of data distribution was analyzed by the Shapiro–Wilk test. For two group comparison, we used Student's t test to analyze if normally distributed continuous variables, otherwise use Mann–Whitney test. For multiple comparison, the data were analyzed by one-way analysis of variance (ANOVA) followed by Bonferroni’s post hoc test if data were normally distributed or by the Kruskal–Wallis test if the data were non-normally distributed. A statistically significant difference was established at *p* < 0.05.

## Supplementary Information

**Additional file 1: Fig. S1**A-D, Behavior tests showing the effects of PTX (50 mg/kg and 100 mg/kg) on BCAS models. A, The percentage of spontaneous alternations in the Y-maze test (*n* ≥ 7 mice per group). B, C, D, The number of platform crossing (*n* ≥ 11 mice per group), the time in target quadrant (*n* = 10 mice per group) of the probe test (Day 6) and escape latency during the acquisition phase (Day 1‐5) (*n* ≥ 10 mice per group). E&F, Immunostaining of Iba-1 (red)/ MBP (green)/ Mertk (gray)/ DAPI (blue) in IC at Day 30 after BCAS. Images of Fig. S2F is 2.5 times magnification of Fig. S2E. Scale bar: 40 μm. All data were presented as the mean ± SEM. *p < 0.05, **p < 0.01, "ns" means no significance (P > 0.05).

**Additional file 2: Fig. S2**A, Quantitative RT-PCR analysis of the expression of TNF-α in IC at Day 30 after BCAS (*n* ≥ 3 mice per group). B, Representative immunoblots probed with antibodies against PPAR-γ, GADPH and H3 in nucleus and cytoplasm of primary microglia. C, Immunofluorescent images of Iba-1 (green)/ PPAR-γ (red)/ DAPI (blue) colocalization in myelin stimulated microglia. Scale bar: 10 μm. D, Quantification of immunofluorescent intensity of PPAR-γ in DAPI area (*n* = 5 repeats per group). E, Verification of specific Mertk knockout in microglia of Cx3cr1-Cre: Mertk fl/fl mice. Immunofluorescent images of Iba-1 (red)/ Mertk (green)/ DAPI (blue) colocalization in IC in of Mertk knockout mice and wild-type littermate. Scale bar: 40 μm. F-K, Behavior tests between intact Mertk cKO mice and their wild littermates. The total moved distance (F) (*n* = 10 mice per group), time percentage in the corner (G) (*n* = 10 mice per group) and time percentage in the center (H) (*n* ≥ 8 mice per group) by open field test. I&J, The exploratory preference to novel objects (*n* = 9 mice per group) in the novel object recognition. K, The percentage of spontaneous alternations in the Y-maze test (*n* = 10 mice per group). All data were presented as the mean ± SEM. *p < 0.05, **p < 0.01, **** < 0.001, "ns" means no significance (P > 0.05).

**Additional file 3: Fig. S3**A&B, Verification of specific Mertk knockout in microglia of Cx3cr1-Cre: Mertk ^fl/fl^ mice. One band represents homozygosity. Two bands represent heterozygosity. A, Image of gel electrophoresis of Mertk. B, Image of gel electrophoresis of Cx3cr1-Cre. C, Gating strategy of flow cytometry. Strategy for cell sorting to obtain FVS-780^low^ viable cells and CD45^int^CD11b^+^ microglia.

## Data Availability

The datasets supporting the conclusions of this article are included in the manuscript.

## Abbreviations

AMP: Adenosine 5′-monophosphate
BCAS: Bilateral common carotid artery stenosis
CCAs: Common carotid arteries
CC: Corpus callosum
cKO: Conditional knockout
CNS: Central nervous system
CSVD: Cerebral small vessel disease
FA: Fractional anisotropy
FBS: Fetal bovine serum
IC: Internal capsule
IL-1β: Interleukin-1β
Mertk: Mer receptor tyrosine kinase
MRI: Magnetic resonance imaging
MS: Multiple sclerosis
MWM: Morris water maze
PBS: Phosphate buffer solution
PPAR: Peroxisome proliferator-activated receptor
PTX: Pentoxifylline
TAM: Tyro3, Axl and Mertk
TNF-α: Tumor necrosis factor-α
VAD: Vascular dementia
WT: Wild-type
