# Supplementary figures and images for: Pentoxifylline alleviates ischemic white matter injury through up-regulating Mertk-mediated myelin clearance
Source: J Neuroinflammation. 2022 May 31;19:128. doi: 10.1186/s12974-022-02480-4 (PMC9153105; doi:10.1186/s12974-022-02480-4)

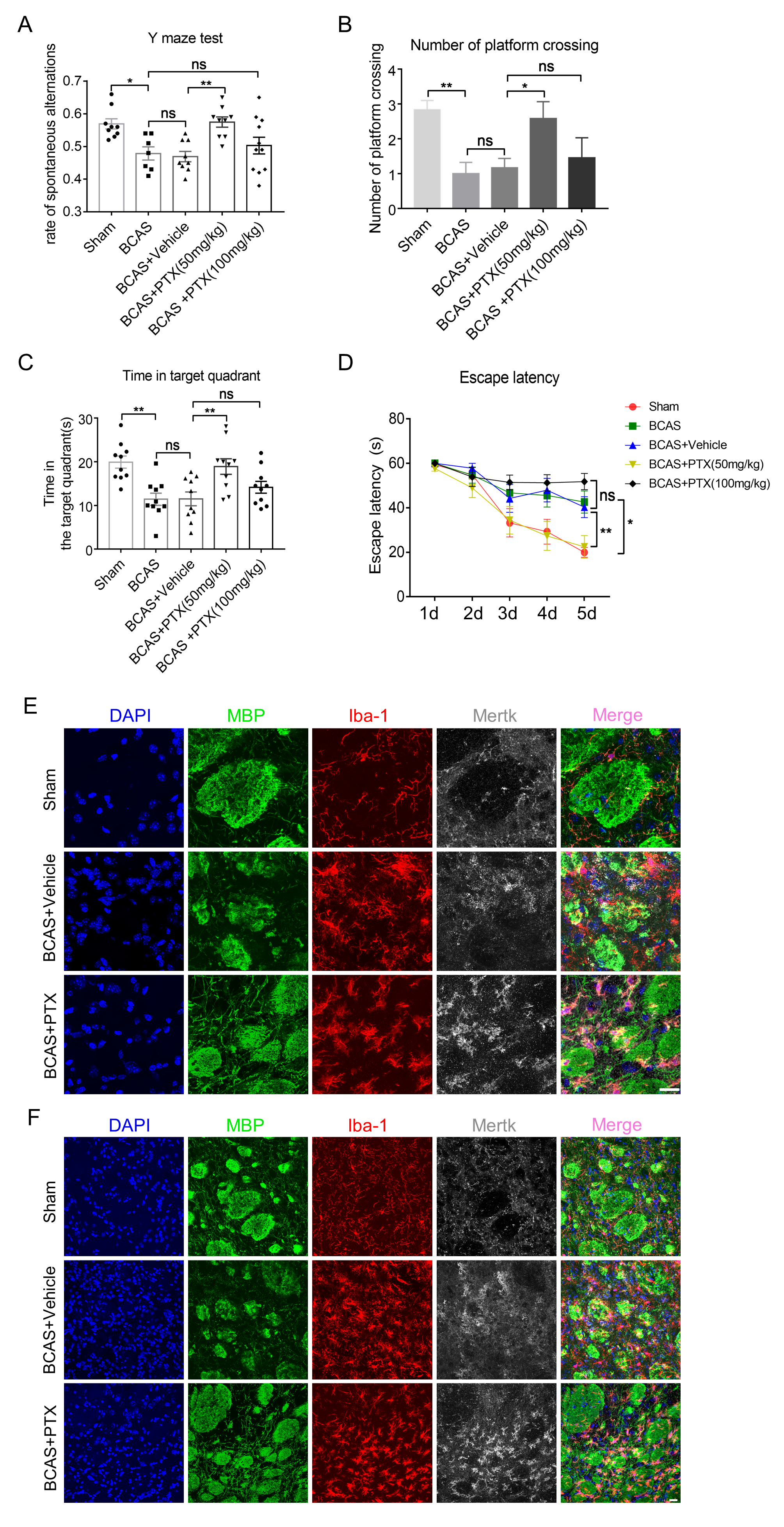

Supplement: Supplementary file 1 — Additional file 1: Fig. S1A-D, Behavior tests showing the effects of PTX (50 mg/kg and 100 mg/kg) on BCAS models. A, The percentage of spontaneous alternations in the Y-maze test (n ≥ 7 mice per group). B, C, D, The number of platform crossing (n ≥ 11 mice per group), the time in target quadrant (n = 10 mice per group) of the probe test (Day 6) and escape latency during the acquisition phase (Day 1‐5) (n ≥ 10 mice per group). E&F, Immunostaining of Iba-1 (red)/ MBP (green)/ Mertk (gray)/ DAPI (blue) in IC at Day 30 after BCAS. Images of Fig. S2F is 2.5 times magnification of Fig. S2E. Scale bar: 40 μm. All data were presented as the mean ± SEM. *p < 0.05, **p < 0.01, "ns" means no significance (P > 0.05). [file 12974_2022_2480_MOESM1_ESM.tif]

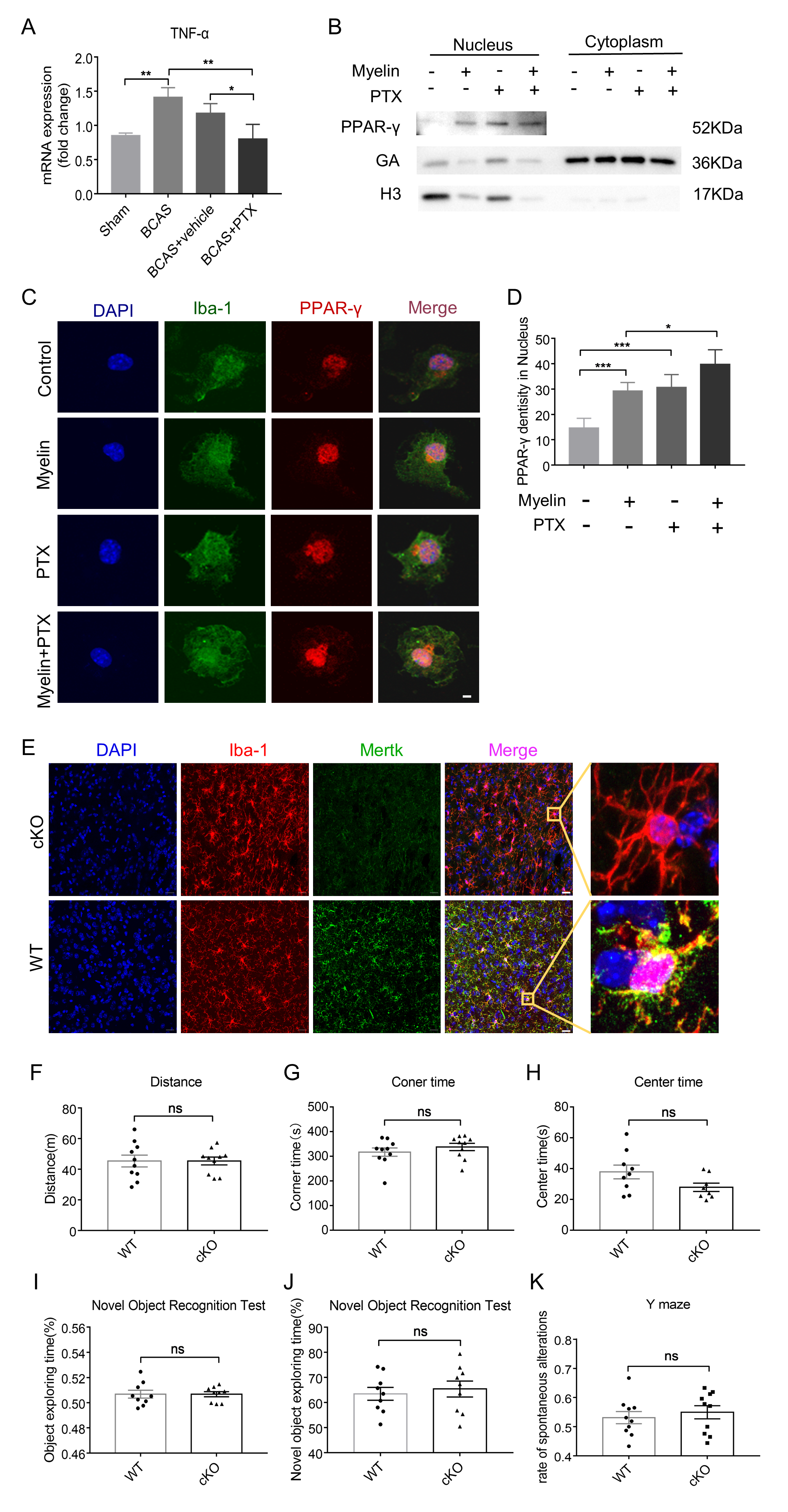

Supplement: Supplementary file 2 — Additional file 2: Fig. S2A, Quantitative RT-PCR analysis of the expression of TNF-α in IC at Day 30 after BCAS (n ≥ 3 mice per group). B, Representative immunoblots probed with antibodies against PPAR-γ, GADPH and H3 in nucleus and cytoplasm of primary microglia. C, Immunofluorescent images of Iba-1 (green)/ PPAR-γ (red)/ DAPI (blue) colocalization in myelin stimulated microglia. Scale bar: 10 μm. D, Quantification of immunofluorescent intensity of PPAR-γ in DAPI area (n = 5 repeats per group). E, Verification of specific Mertk knockout in microglia of Cx3cr1-Cre: Mertk fl/fl mice. Immunofluorescent images of Iba-1 (red)/ Mertk (green)/ DAPI (blue) colocalization in IC in of Mertk knockout mice and wild-type littermate. Scale bar: 40 μm. F-K, Behavior tests between intact Mertk cKO mice and their wild littermates. The total moved distance (F) (n = 10 mice per group), time percentage in the corner (G) (n = 10 mice per group) and time percentage in the center (H) (n ≥ 8 mice per group) by open field test. I&J, The exploratory preference to novel objects (n = 9 mice per group) in the novel object recognition. K, The percentage of spontaneous alternations in the Y-maze test (n = 10 mice per group). All data were presented as the mean ± SEM. *p < 0.05, **p < 0.01, **** < 0.001, "ns" means no significance (P > 0.05). [file 12974_2022_2480_MOESM2_ESM.tif]

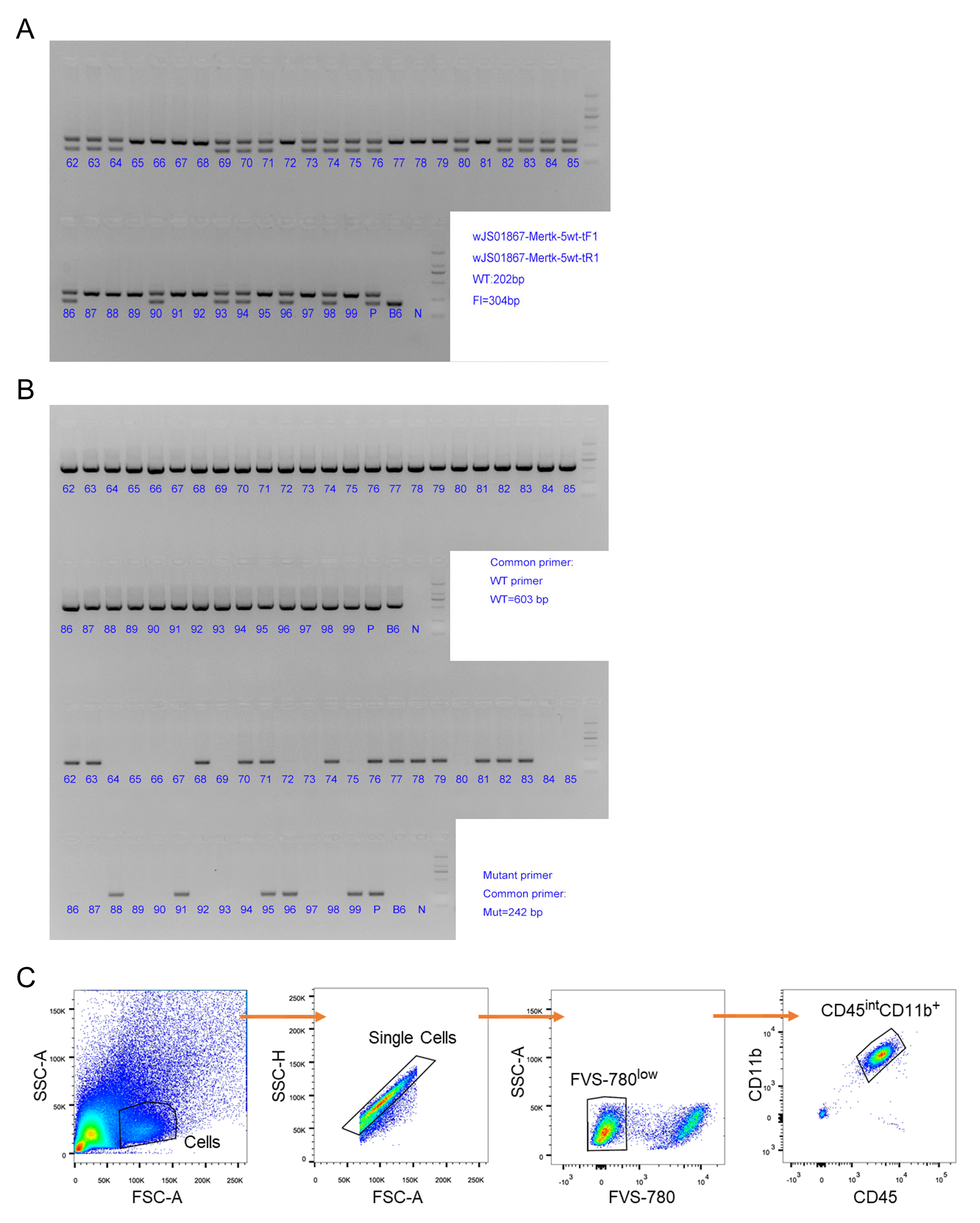

Supplement: Supplementary file 3 — Additional file 3: Fig. S3A&B, Verification of specific Mertk knockout in microglia of Cx3cr1-Cre: Mertk fl/fl mice. One band represents homozygosity. Two bands represent heterozygosity. A, Image of gel electrophoresis of Mertk. B, Image of gel electrophoresis of Cx3cr1-Cre. C, Gating strategy of flow cytometry. Strategy for cell sorting to obtain FVS-780low viable cells and CD45intCD11b+ microglia. [file 12974_2022_2480_MOESM3_ESM.tif]
